# What is the advance of extent of resection in glioblastoma surgical treatment—a systematic review

**DOI:** 10.1186/s41016-018-0150-7

**Published:** 2019-02-01

**Authors:** Lei Wang, Buqing Liang, Yan Icy Li, Xiang Liu, Jason Huang, Yan Michael Li

**Affiliations:** 1 Department of Neurosurgery and Radiology, University of Rochester Medical Center, 601 Elmwood Avenue, Rochester, NY 14642 USA; 2 Department of Neurosurgery, Baylor Scott & White Health, Temple, TX 76508 USA; 3 Department of Neurosurgery, The Affiliated Hospital of Xuzhou Medical University, 99 Huaihai West Road, Xuzhou, 221002 Jiangsu Province China; 4 Department of Bioinformatics, Nanjing Medical University, Nanjing, 211166 China

**Keywords:** Glioblastoma multiform (GBM), Biopsy, Extent of resection (EOR), Gross total resection (GTR)

## Abstract

Glioblastoma multiform (GBM) is the most common malignant brain tumor characterized by poor prognosis, increased invasiveness, and high relapse rates. The relative survival estimates are quite low in spite of the standard treatment for GBM in recent years. Now, it has been gradually accepted that the amount of tumor mass removed correlates with longer survival rates. Although new technique advances allowing intraoperative analysis of tumor and normal brain tissue and functional paradigms based on stimulation techniques to map eloquent areas have been used for GBM resection, visual identification of tumor margins still remains a challenge for neurosurgeons. This article attempts to review and summarize the evolution of surgical resection for glioblastomas.

## Background

Glioblastoma multiform (GBM) is the most common of all malignant central nervous system (CNS) tumors, which accounts for 47.1% of primary malignant brain tumors, or about 12,390 new cases each year in the USA [1]. Since GBM is one of the most aggressive types of cancer, relative survival estimates are quite low, with only 5.1% of patients surviving 5 years after diagnosis [2]. The optimal treatment for GBM consists of the combination of surgical resection, radiation therapy, and chemotherapy [3–5]. The surgical resection for glioma aims at relieving mass effect, achieving cytoreduction, and providing adequate tissue for histology and molecular tumor characterization [3]. In the absence of new neurological deficit, which are one of the prognostic factors in glioblastomas, higher volume of tumor mass resected correlates with longer survival in GBM patients [6, 7]. Furthermore, there is no controversy about maximal resection which is beneficial for patient’s prognosis compared to biopsy or no surgery. In general, there are three stages in the development of surgical treatment for GBM: (1) early biopsy, (2) gross total resection (GTR) for contrast-enhanced T1 magnetic resonance imaging (MRI), and (3) Maximal-resection for T2 magnetic resonance imaging or fluid-attenuated inversion recovery (FLAIR) abnormalities. This article reviews the key historic literature and focuses on the evolution of surgical resection strategies for glioblastomas.

## Early biopsy stage

One of the founding fathers of modern neurosurgery, Walter Dandy [8], described the five right hemispherectomy procedures that he performed for the treatment of gliomas. Though he briefly mentioned the pathologies for some of the cases, it is safe to judge from his description that a number of tumors possessed the characteristics of malignancy: spreading to the contralateral side via corpus callosum, and recurrence in a short period of time. In the conclusion part, Dandy concluded that although this was a very aggressive operation to be advised, it nevertheless offers much longer extension of life to patients compared to any other possible form of treatment. Moreover, when the tumor cannot be completely resected, the procedure offers a treatment possibility otherwise not obtainable in certain tumors situated within the confines of the hemisphere. This can be considered as the first documented surgical procedure proposed for the treatment of malignant gliomas.

## Contrast-enhanced T1 MRI for gross total resection

With the advent of computed tomography (CT) and magnetic resonance imaging (MRI), neurosurgeons were able to localize the tumor preoperatively, dramatically improving surgical accuracy and lowering morbidity and mortality. Moreover, the amount of tumor mass removed was no longer an approximate estimate of the neurosurgeons. Lacroix et al. [9] introduced the novel concept of a maximum extent of resection (EOR). The study reported the result of a retrospective study based on a cohort of 416 patients with histological proven GBM who underwent tumor resection and identified five prognostic predictors including age, Karnofsky Performance Scale (KPS) score, extent of resection, and the degree of necrosis and enhancement on preoperative MRI. Furthermore, they reached the conclusion that resection of 89% or more of the tumor volume was necessary to obtain significant survival improvement after surgery, while resection of 98% or more of the tumor volume is a significant independent predictor of survival in the multivariate analysis for the whole group. This paper established the concept that the highest tumor volume resected leads to better prognosis in GBM. Specifically, resection of 89% or more of the tumor volume significantly increases patient’s survival. Ten years later, Sanai et al. [10] looked at a cohort of 500 newly diagnosed supratentorial GBM patients between 1997 and 2005 who underwent surgical resection of the tumors followed by standard chemotherapy and radiation therapy. Patients that had previous resection or neoadjuvant therapy were excluded. Interestingly, it was demonstrated that as little as 78% EOR resulted in a significant survival advantage. At almost the same time, Orringer et al. [11] showed that EOR is significantly affected by tumor location, size, and neurosurgeon’s experience. This retrospective study also revealed a relationship between EOR and survival indicating that EOR greater than 90% was associated with greater 1-year survival than EOR less than 90%. However, it was objected that the mixed patient sampling of both newly diagnosed and recurrent GBM disrupted the uniformity of the study.

The concept of maximum EOR stimulated active research and leads to several other publications on the relationship among gross total resection (GTR), residual volume (RV), and survival [4, 12–17]. Most recently, the first quantitative meta-analysis on association of the extent of resection with survival in glioblastoma was carried out by Brown et al. [18] who systematically reviewed relevant papers published from January 1996 to December 2015. Adult patients (37 studies were recruited) with newly diagnosed supratentorial GBM undergoing various EORs were analyzed and presented objective overall or progression-free survival (PFS) data. The meta-analysis revealed that GTR probably increases the likelihood of 1-year survival compared with subtotal resection (STR) by about 61%, 2-year survival by about 19%, and progression-free survival at 12 months by 51%. In conclusion, GTR is favored in all patients with newly diagnosed GBM.

## T2 MRI or FLAIR for maximal resection

Sherriff et al. [19] published a retrospective study to discuss the optimal irradiation pattern for postoperative patients with glioblastoma and suggested that most of relapsing tumors occur within 2 cm of the original contrast-enhanced mass. Due to the invasive nature of glioblastoma, it is not surprising that the tumor cells have already infiltrated beyond the contrast-enhanced T1 MRI. Here comes a question: can we do better than gross total resection? When most of neurosurgeons focused on gross total resection of contrast-enhanced T1 MRI, Li et al. [20] pushed the boundary of surgical resection procedures even further by performing additional resection of the T2 MRI or FLAIR abnormality beyond the contrast-enhanced T1 MRI. Their conclusion consists of two parts: (1) The median survival time for complete resection patients was significantly longer than that for incomplete resection patients (15.2 months versus 9.8 months, *p* < 0.001). (2) Resection of a significant portion (≥ 53.21%) of the T2 MRI or FLAIR abnormality region, if feasible and safely attempted, could have a beneficial impact on the survival of patients with GBM (median survival time 20.7 months versus 15.5 months, *p* < 0.001). At the same time, Brian et al. [21] demonstrated that the molecular and cellular composition of nonenhanced (NE) region differ significantly from those of the contrast-enhanced (CE) regions of GBM as determined by radiographically localized biopsies. CE samples had significantly higher cellularity than NE samples. The NE region showed the histological features of diffusely infiltrating glioma with neoplastic glial cells intermingled with nonneoplastic and reactive cells. This novel abnormal T2 MRI or FLAIR resection can be used to identify and access new therapeutic targets that may be expressed by infiltrating glioma cells or by nonneoplastic/reactive cells that play a key role in disease progression and stimulate further investigations.

## Discussion

Dating back to Dandy’s era, investigations on the best surgical procedures to maximize treatment outcome in gliomas have been spanning for almost 100 years. Based on the concept of obtaining tissue sample for definitive diagnosis and reducing intracranial pressure, biopsy remained the main purpose of surgery in the middle to late twentieth century which was performed by either burr-hole or craniotomy [22–24]. As more evidence surfaced supporting the positive correlation between resection and survival, gross total resection came to the scientific community attention. Now, maximal safe resection of malignant gliomas as the first step of standard therapy is an accepted treatment strategy in malignant glioma surgery, and more evidence has elucidated a positive relationship between surgical resection and survival [18, 20, 25, 26]. In recent years, some new tools and techniques have been implemented to safely achieve GTR and to improve surgical results, such as fluorescein-guided technique, ultrasonography, intraoperative MRI (iMRI), and neuronavigation with functional MRI (fMRI). In the past 2 years, fluorescein sodium- and 5-ALA-guided techniques have been reported in many institutions to be effective for maximal safe resection of GBM and for prolonging the patients’ progression-free survival. Moreover, some reports supported the notion that these tools have an effect also on the patients’ overall survival (OS). Although growing evidence supported the use of intraoperative fluorescent agents, there are some hindered drawbacks for these techniques. To date, 5-ALA remains an expensive compound and requires special equipment and environment during the surgical procedure, although specificity and sensitivity for tumoral tissues of 5-ALA are 100% and 85% respectively [27], and fluorescein sodium has been based only on observational cohort studies and case series [16, 27].

Similar to 5-ALA-guided resection results, the use of preoperative fMRI and neuronavigation have improved maximal safe resection of GBM and prolonged median survival time to 20.7 months in our previously published article [20]. However, brain shift is an intrinsic difficulty in the use of neuronavigation, which result from patient’s positioning, dural opening, cerebrospinal fluid (CSF) loss, residual tumor volume decreasing, and peritumoral edema. The technique of iMRI offers a unique method to monitor brain shift, register whenever necessary, and to quantitative the residual tumoral volume, then using this data to decide whether further resection is necessary or not. Some authors have demonstrated that the use of iMRI achieved higher rate of GTR and significantly improved survival compared with conventional surgery using intraoperative neuronavigation alone [28–31]. Furthermore, Coburger et al. [32] even found that combining 5-ALA with iMRI achieved GTR in 100% of patients compared with 82% when using iMRI alone. Eyupoglu et al. [33] also demonstrated that combining these two techniques of iMRI and 5-ALA significantly improved life expectancy (from 14 to 18.5 months) for “supra-complete resection.” However, some authors argued that MRI only provided tumor anatomic detail and localization, which have limited utility in delineating the full tumor contour. Ellen et al. [34] described a novel technique combining positron emission tomography (PET)-guided MR spectroscopy (MRS). This study introduced MRS, which was initially non-diagnostic for malignancy, as a tool to diagnose grade IV GBM when combined with PET. Benoit et al. [35] also reported that metabolic information helped the surgical planning and PET-guided resection resulted in longer survival in GBM, while MRI-guided was not correlated with a significantly better survival. Limited data is available regarding the OS and the quality of life of patients, both of which are more of a concern for neurosurgeons. Although iMRI seems to have a better application prospect, high cost equipment, operating room refurbishment, and longer operating time limits its widespread use.

Intraoperative ultrasound is relatively inexpensive tool compared to iMRI and is convenient to account for brain shift, predict residual tumor, and visualize vascular relationships to tumor. Some authors have demonstrated that GTR was achieved in 55%–83% of their cases with or without neuronavigation when complete resection was the goal of surgery [36–38]. Prada et al. [38] recently reported that intraoperative ultrasound have an effective and specific role in identifying residual tumor, especially using contrast-enhanced ultrasound in GBM surgery. In a meta-analysis, Mahboob et al. [39] found an average GTR rate of 71% for high-grade gliomas compared to other techniques. However, there are still some drawbacks. Intraoperative ultrasound is an operator-dependent technique, so the learning curve for the technique can be steep and the level of experience in its use can affect image quality, orientation, and interpretation. Furthermore, different surface, bleeding, and hemostatic agents during the surgical procedure affect image quality, sensitivity, and specificity, which influence the decision to proceed with further resection. Despite these drawbacks, the development of 3D and higher frequency cranial probes still makes this technology attractive for the removal of malignant gliomas in the future.

When identifying eloquent areas of GBM involving cortical and subcortical regions, emerging technologies such as awake craniotomy (AC), laser interstitial thermal therapy (LITT), and Raman spectroscopy seem to be more effective in removing tumors without affecting the patient’s quality of life. The advantages of AC include better GTR, improved postoperative functional status, better postoperative KPS status, lower length of hospitalization, and low postoperative morbidities, especially when the tumor located in eloquent areas where the neurosurgeon cannot rely on the MRI and the anatomy itself for the maximal extent of resection [40–44]. LITT has been used to treat glioblastoma which was difficult to resect [45], and recurrent GBM [46]. All patients treated with LITT showed a significant increase in overall survival compared to traditional therapy. Jermyn et al. [47] developed a handheld probe (Raman spectroscopy) to differentiate between normal white and grey matter in tumor tissue, and obtained a sensitivity of 94% and specificity of 91% for WHO grade IV glioblastoma tissue. Banerjee and Verma [48] highly endorsed the application prospect of this technology in glioma. However, only few medical centers reported that LITT and Raman spectroscopy are effective in GBM therapy, calling for more random trials which are necessary to evaluate the results of LITT in the future.

Apart from surgical treatment strategies, adjuvant therapies including chemotherapy and radiotherapy should be considered in the standard of care for GBM patients as they also influence patient survival, PFS, and OS [5, 49–51]. Matsuda et al. [25] reported a median OS of 36.9 months for patients who underwent GTR of newly diagnosed GBM, with a particular focus on the influence of the subventricular zone (SVZ), combined with high-dose proton beam therapy, compared with a median OS of 26.2 months for patients treated with conventional radiotherapy. Emmanuel et al. [52] evaluated GBM patients characterized by long-term survival (LTS; survival of at least 3 years after diagnosis). Twelve patients out of 101 that underwent surgical resection became LTS patients. Among those 12 LTS patients, seven of them had a gross total resection (GTR), including two with an additional resection after iMRI, three had a near total resection and one had a partial resection, ten patients had a methylation of methylguanine-DNA methyltransferase (MGMT), only two had an isocitrate dehydrogenase 1 (IDH1) mutation, and seven received a full Stupp protocol. Half of the patients with a second surgery survived at least 2 years postoperatively. Those encouraging observations emphasize the importance of maximizing the resection using advanced techniques, and future research should focus on the microenvironment of GBM and, if necessary, multiple surgical procedures although GBM therapy remains challenging.

## Abbreviations

5-ALA: 5-Aminolevulinic acid
AC: Awake craniotomy
CE: Contrast-enhanced
CNS: Central nervous system
CSF: Cerebrospinal fluid
CT: Computed tomography
EOR: Extent of resection
FLAIR: Fluid-attenuated inversion recovery
fMRI: Functional magnetic resonance imaging
GTR: Gross total resection
IDH: Isocitrate dehydrogenase
iMRI: Intraoperative magnetic resonance imaging
KPS: Karnofsky Performance Scale
LITT: Laser interstitial thermal therapy
LTS: Long-term survival
MGMT: Methylguanine-DNA methyltransferase
MRI: Magnetic resonance imaging
NE: Nonenhanced
OS: Overall survival
PFS: Progression-free survival
RV: Residual volume
STR: Subtotal resection
SVZ: Subventricular zone
